# Supplementary material for: Strengthening laboratories in response to outbreaks in humanitarian emergencies and conflict settings: Results, challenges and lessons from expanding PCR diagnostic capacities for COVID-19 testing in Yemen
Source: PLoS One. 2024 Feb 23;19(2):e0298603. doi: 10.1371/journal.pone.0298603 (PMC10889613; doi:10.1371/journal.pone.0298603)
Supplement: Attachment — Submitted filename: Response to reviewers.docx [file pone.0298603.s002.docx]

Rebuttal letter

Dear Mathumalar Loganathan Fahrni- Academic Editor - PLOS ONE

I would like to say ‘’thank you’’ to the feedback on the manuscript from the journal.

I have taken note of the valuable comments suggested by the different editors, the editorial team, and reviewers and I have attempted to provide the required feedback.

In the email received from the editor, I have attempted to give my response under each comment and highlighted with yellow colour.

In summary,

- I have changed the format of the manuscript to comply with PLOS editorial policy
- I have attached the underlying data sets for the manuscript
- In regards to Fig 1 and Fig 4, I have attached signed approval from the party with copyright
- We have commented on the advice from the editorial team on the change of format.
- I have reviewed the list of the references and I did not find any article that has been withdrawn or no longer available – if the editorial team finds any article that is no longer available, I will appreciate if that particular reference is highlighted.
- I have responded to the comments from the two reviewers – where comment was necessary.

I hope that you will be able to find our response and comments sufficient to the queries raised by the different reviewers. Moreover, we will be happy and will be more obliged to respond to any further questions and clarification if and when required.

Thank you and regards.

Ismail M Bashir

Dear Dr. BASHIR,

Thank you for submitting your manuscript to PLOS ONE. After careful consideration, we feel that it has merit but does not fully meet PLOS ONE’s publication criteria as it currently stands. Therefore, we invite you to submit a revised version of the manuscript that addresses the points raised during the review process.

Please submit your revised manuscript by Dec 01 2023 11:59PM. If you will need more time than this to complete your revisions, please reply to this message or contact the journal office at [plosone@plos.org](mailto:plosone@plos.org). When you're ready to submit your revision, log on to <https://www.editorialmanager.com/pone/> and select the 'Submissions Needing Revision' folder to locate your manuscript file.

Please include the following items when submitting your revised manuscript:

- A rebuttal letter that responds to each point raised by the academic editor and reviewer(s). You should upload this letter as a separate file labeled 'Response to Reviewers'.
- A marked-up copy of your manuscript that highlights changes made to the original version. You should upload this as a separate file labeled 'Revised Manuscript with Track Changes'.
- An unmarked version of your revised paper without tracked changes. You should upload this as a separate file labeled 'Manuscript'.

If you would like to make changes to your financial disclosure, please include your updated statement in your cover letter. Guidelines for resubmitting your figure files are available below the reviewer comments at the end of this letter.

If applicable, we recommend that you deposit your laboratory protocols in protocols.io to enhance the reproducibility of your results. Protocols.io assigns your protocol its own identifier (DOI) so that it can be cited independently in the future. For instructions see: <https://journals.plos.org/plosone/s/submission-guidelines#loc-laboratory-protocols>. Additionally, PLOS ONE offers an option for publishing peer-reviewed Lab Protocol articles, which describe protocols hosted on protocols.io. Read more information on sharing protocols at <https://plos.org/protocols?utm_medium=editorial-email&utm_source=authorletters&utm_campaign=protocols>.

We look forward to receiving your revised manuscript.

Kind regards,

Mathumalar Loganathan Fahrni
Academic Editor
PLOS ONE

Journal Requirements:

When submitting your revision, we need you to address these additional requirements.

1. Please ensure that your manuscript meets PLOS ONE's style requirements, including those for file naming. The PLOS ONE style templates can be found at
   <https://journals.plos.org/plosone/s/file?id=wjVg/PLOSOne_formatting_sample_main_body.pdf> and <https://journals.plos.org/plosone/s/file?id=ba62/PLOSOne_formatting_sample_title_authors_affiliations.pdf>

Revised according to the PLOS ONE's style requirements

2. In your Data Availability statement, you have not specified where the minimal data set underlying the results described in your manuscript can be found. PLOS defines a study's minimal data set as the underlying data used to reach the conclusions drawn in the manuscript and any additional data required to replicate the reported study findings in their entirety. All PLOS journals require that the minimal data set be made fully available. For more information about our data policy, please see <http://journals.plos.org/plosone/s/data-availability>.

""Upon re-submitting your revised manuscript, please upload your study’s minimal underlying data set as either Supporting Information files or to a stable, public repository and include the relevant URLs, DOIs, or accession numbers within your revised cover letter. For a list of acceptable repositories, please see <http://journals.plos.org/plosone/s/data-availability#loc-recommended-repositories>. Any potentially identifying patient information must be fully anonymized.

Important: If there are ethical or legal restrictions to sharing your data publicly, please explain these restrictions in detail. Please see our guidelines for more information on what we consider unacceptable restrictions to publicly sharing data: <http://journals.plos.org/plosone/s/data-availability#loc-unacceptable-data-access-restrictions>. Note that it is not acceptable for the authors to be the sole named individuals responsible for ensuring data access.

We will update your Data Availability statement to reflect the information you provide in your cover letter.

Minimal underlying data set submitted as supporting information and is attached

3. We note that Figures 1 and 4 in your submission contain [map/satellite] images which may be copyrighted. All PLOS content is published under the Creative Commons Attribution License (CC BY 4.0), which means that the manuscript, images, and Supporting Information files will be freely available online, and any third party is permitted to access, download, copy, distribute, and use these materials in any way, even commercially, with proper attribution. For these reasons, we cannot publish previously copyrighted maps or satellite images created using proprietary data, such as Google software (Google Maps, Street View, and Earth). For more information, see our copyright guidelines: <http://journals.plos.org/plosone/s/licenses-and-copyright>.
    
   We require you to either (1) present written permission from the copyright holder to publish these figures specifically under the CC BY 4.0 license, or (2) remove the figures from your submission:
    
   a. You may seek permission from the original copyright holder of Figures 1 and 4 to publish the content specifically under the CC BY 4.0 license.  
    
   We recommend that you contact the original copyright holder with the Content Permission Form (<http://journals.plos.org/plosone/s/file?id=7c09/content-permission-form.pdf>) and the following text:
   “I request permission for the open-access journal PLOS ONE to publish XXX under the Creative Commons Attribution License (CCAL) CC BY 4.0 (<http://creativecommons.org/licenses/by/4.0/>). Please be aware that this license allows unrestricted use and distribution, even commercially, by third parties. Please reply and provide explicit written permission to publish XXX under a CC BY license and complete the attached form.”
    
   Please upload the completed Content Permission Form or other proof of granted permissions as an ""Other"" file with your submission.
    
   In the figure caption of the copyrighted figure, please include the following text: “Reprinted from [ref] under a CC BY license, with permission from [name of publisher], original copyright [original copyright year].”
    
   b. If you are unable to obtain permission from the original copyright holder to publish these figures under the CC BY 4.0 license or if the copyright holder’s requirements are incompatible with the CC BY 4.0 license, please either i) remove the figure or ii) supply a replacement figure that complies with the CC BY 4.0 license. Please check copyright information on all replacement figures and update the figure caption with source information. If applicable, please specify in the figure caption text when a figure is similar but not identical to the original image and is therefore for illustrative purposes only.
   The following resources for replacing copyrighted map figures may be helpful:
    
   USGS National Map Viewer (public domain): <http://viewer.nationalmap.gov/viewer/>
   The Gateway to Astronaut Photography of Earth (public domain): <http://eol.jsc.nasa.gov/sseop/clickmap/>
   Maps at the CIA (public domain): <https://www.cia.gov/library/publications/the-world-factbook/index.html> and <https://www.cia.gov/library/publications/cia-maps-publications/index.html>
   NASA Earth Observatory (public domain): <http://earthobservatory.nasa.gov/>
   Landsat: <http://landsat.visibleearth.nasa.gov/>
   USGS EROS (Earth Resources Observatory and Science (EROS) Center) (public domain): [http://eros.usgs.gov/#](http://eros.usgs.gov/)
   Natural Earth (public domain): <http://www.naturalearthdata.com/>

A permission from the original copyright holder of Figures 1 and 4 – attached as other documents

4. Please review your reference list to ensure that it is complete and correct. If you have cited papers that have been retracted, please include the rationale for doing so in the manuscript text, or remove these references and replace them with relevant current references. Any changes to the reference list should be mentioned in the rebuttal letter that accompanies your revised manuscript. If you need to cite a retracted article, indicate the article’s retracted status in the References list and also include a citation and full reference for the retraction notice.

   Additional Editor Comments:

   Dear Authors,

   The manuscript highlights the increasing need for molecular-based diagnostics and the processes which Yemen had underwent to achieve greater capacity for laboratory testing.

   Could the authors kindly resubmit after writing the assessment report using the format of a strengths–weaknesses–opportunities–threats (SWOT) analytical method.

   Regards,

   Plos One Editorial Team

   We acknowledge the current format of the report may seem suitable to present it using SWOT methodology. We believe that we will have another opportunity to perform the SWOT assessment of the laboratory systems in Yemen. However, for the current report we prefer to retain in its current format as an article.

[Note: HTML markup is below. Please do not edit.]

Reviewers' comments:

Reviewer's Responses to Questions

**Comments to the Author**

1. Is the manuscript technically sound, and do the data support the conclusions?

The manuscript must describe a technically sound piece of scientific research with data that supports the conclusions. Experiments must have been conducted rigorously, with appropriate controls, replication, and sample sizes. The conclusions must be drawn appropriately based on the data presented.

Reviewer #1: Yes

Reviewer #2: Yes

2. Has the statistical analysis been performed appropriately and rigorously?

Reviewer #1: Yes

Reviewer #2: Yes

3. Have the authors made all data underlying the findings in their manuscript fully available?

The [PLOS Data policy](http://www.plosone.org/static/policies.action#sharing) requires authors to make all data underlying the findings described in their manuscript fully available without restriction, with rare exception (please refer to the Data Availability Statement in the manuscript PDF file). The data should be provided as part of the manuscript or its supporting information, or deposited to a public repository. For example, in addition to summary statistics, the data points behind means, medians and variance measures should be available. If there are restrictions on publicly sharing data—e.g. participant privacy or use of data from a third party—those must be specified.

Reviewer #1: Yes

Reviewer #2: Yes

4. Is the manuscript presented in an intelligible fashion and written in standard English?

PLOS ONE does not copyedit accepted manuscripts, so the language in submitted articles must be clear, correct, and unambiguous. Any typographical or grammatical errors should be corrected at revision, so please note any specific errors here.

Reviewer #1: Yes

Reviewer #2: Yes

5. Review Comments to the Author

Please use the space provided to explain your answers to the questions above. You may also include additional comments for the author, including concerns about dual publication, research ethics, or publication ethics. (Please upload your review as an attachment if it exceeds 20,000 characters)

Reviewer #1: This study aimed to evaluate the laboratory capacity of the Republic of Yemen before and after the Corona virus
117 Using a number of parameters including the number of facilities with technical capacity for PCR, PCR
Output of 118 tests daily, staff training/proficiency in PCR, utilization rates, no
119 cases/diseases diagnosed by PCR and others. Timetables and important milestones in
120 projects and in the COVID-19 pandemic highlighted the ways in which laboratory preparedness and
Strategic selection of 121 laboratories helped identify the index case of COVID-19 in Yemen. The article is very clear and has importance for Yemen and even for the world in clarifying the technical and economic problems facing these countries.

No comments were required

Reviewer #2: The topic included Yemen as a whole, while the laboratories in which Covid tests are available are in some governorates, so why was it not specified in the topic, for example, the main governorates in Yemen?

Comments to reviewer #2: The project intended to support laboratory strengthening in the Yemen as a whole. The names and locations where the services are available are highlighted in the Fig 1. Majority of the governorates either had a PCR laboratory facility and we preferred the main topic to highlight that - covering all Yemen. However, we have included statements on the limitation of the report in regards to the this and specifically highlighting that the data presented were from specific governorates that were submitting data in regards to COVID-19 testing.

6. PLOS authors have the option to publish the peer review history of their article ([what does this mean?](https://journals.plos.org/plosone/s/editorial-and-peer-review-process#loc-peer-review-history)). If published, this will include your full peer review and any attached files.


If you choose “no”, your identity will remain anonymous but your review may still be made public.


**Do you want your identity to be public for this peer review?** For information about this choice, including consent withdrawal, please see our [Privacy Policy](https://www.plos.org/privacy-policy).

Reviewer #1: **Yes:** Hassan Abdulwahab Al-Shamahy

Reviewer #2: No
